# Case Report: Molecular and Pathological Investigations of Zoonotic *Anatrichosoma* Spp.-Induced Ulcerative Pododermatitis in a Domestic Cat in Thailand

**DOI:** 10.3389/fvets.2021.759814

**Published:** 2021-10-14

**Authors:** Wanarit Jitsamai, Sawang Kesdangsakonwut, Thanakorn Srirat, Piyanan Taweethavonsawat

**Affiliations:** ^1^Parasitology Unit, Department of Pathology, Faculty of Veterinary Science, Chulalongkorn University, Bangkok, Thailand; ^2^Pathology Unit, Department of Pathology, Faculty of Veterinary Science, Chulalongkorn University, Bangkok, Thailand; ^3^Faculty of Veterinary Medicine, Veterinary Teaching Hospital, Khon Kean University, Khon Kaen, Thailand; ^4^Biomarkers in Animal Parasitology Research Group, Chulalongkorn University, Bangkok, Thailand

**Keywords:** *Anatrichosoma* spp., pododermatitis, cat, Thailand, molecular investigation

## Abstract

*Anatrichosoma* spp. is a group of trichuroid nematodes that mainly infect non-human primates and domestic cats. The lifecycle of these nematodes remains unclear. In non-human primates, *Anatrichosoma* spp. were found in the nasal cavity. However, ulcerative dermatitis has been reported in infected cats. An adult, intact, female domestic short-haired cat was presented with ulcerative pododermatitis of all limbs. Punch biopsy was performed at the edge of the ulcerative wound for histopathological investigation and showed necrosis and infiltration of inflammatory cells around the nematode-like lesion. Eggs with *Capillaria*-like characteristics were present. Tissue sections were subjected to DNA extraction and PCR targeting *18S rRNA*, using primers designed from *Anatrichosoma 18S rRNA*. The phylogenetic tree revealed that DNA obtained from the lesion of the domestic cat was grouped with *Anatrichosoma* spp. from the olive glass mouse (*Abothirx olivacea*)*, Capillaria plica* and *Eucoleus aerophilus*, both from the red fox (*Vulpes Vulpes*). The study is the first report of feline anatrichosomiasis in Thailand, and we present both pathological findings and molecular evidence. The cat was successfully treated with emodepsine/praziquantel. The skin lesion recovered within 3 days after anthelmintic treatment. Because *Anatrichosoma* spp. have been reported in humans, the zoonotic potential of this parasite should be considered.

## Introduction

*Anatrichosoma* spp. is a genus of trichuroid nematodes and infects a wide range of hosts. Several species are known, including *Anatrichosoma buccalis* (1, 2)*, A. rhina, A. nacepo* (3)*, A. haycocki* (4), *A. ocularis* (5), and *A. cynamolgi* (6, 7). *Anatrichosoma* spp. has also been reported in humans and is considered a zoonotic nematode (8, 9). However, the lifecycle of this parasite is poorly understood. Anatrichosomiasis has been reported in mucosal and submucosal nasal cavities in non-human primates, particularly the rhesus monkey (*Macaca mulatta*) and cynomolgus macaques (*Macaca fascicularis*) in Southeast Asia (3, 10). In white-handed gibbons (*Hylobates lar*), this parasite was found in the ears, lips, nares and eyelids (11). *Anatrichosoma* spp. has also been observed in the paracloacal gland of the dusky antechinus (*Antechinus swainsonii*) and brown antechinus (*A. stuartii*) (4). Human anatrichosomiasis causes multiple oral ulcers and can also be found in the subcutaneous nodule of the breast (8, 9). Opossums (*Didelphis marsupialis* and *D. virginiana*) can be infected with *A. buccalis*, causing pododermatitis; it can also be found in the mucosal palate (1, 2). In the common tree shew (*Tupaia glis*), *Anatrichosoma ocularis* has been detected in the eyes (5). *Anatrichosomiasis* in domestic cats has been reported in Namibia, South Africa and the United States, causing ulcerative pododermatitis (12–14). In dogs, *Anatrichosoma* spp. has been found in the nodule on the dorsal midline in the lumbar region (15).

*Anatrichosoma* spp. infection is typically diagnosed based on pathological lesions. To our knowledge, only one nucleotide sequence of *Anatrichosoma* spp. has been submitted in GenBank (Accession number KU215886). However, a molecular identification protocol has not been published. This study describes, for the first time, *Anatrichosoma* spp.-induced ulcerative pododermatitis in a domestic cat in Thailand, using pathological and molecular data.

## Case Presentation

**Figure 1:**
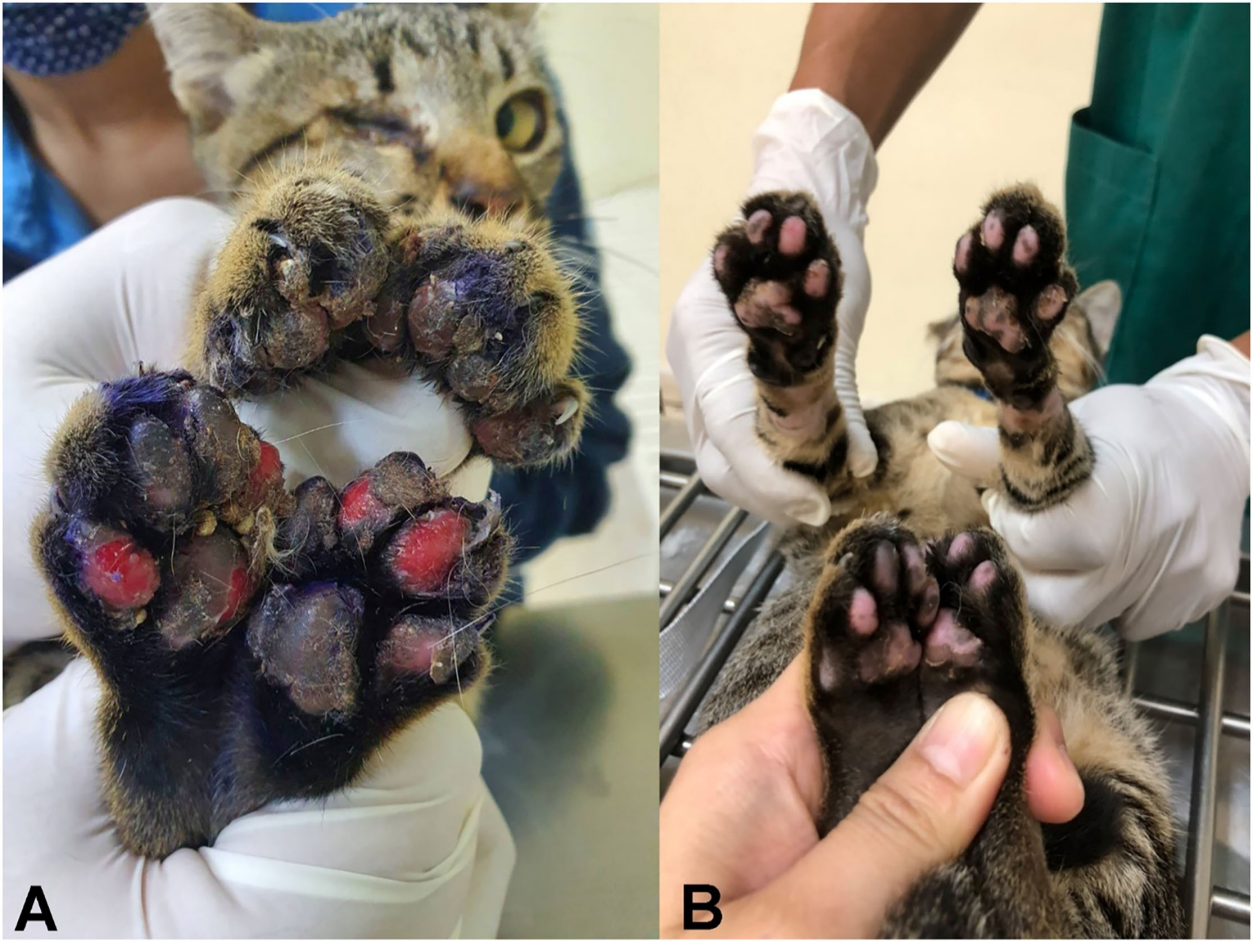
Ulcerative pododermatitis caused by *Anatrichosoma* spp. was presented in four feet **(A)**; 2 weeks post-treatment with emodepsine/praziquantel led to complete recovery **(B)**.

The cat described here was an adult, intact, female domestic short-haired cat. Although it was kept outdoors with other cats, it was the only clinically affected one. The cat was presented to the Veterinary Teaching Hospital, Faculty of Veterinary Medicine, Khon Kean University, because of feet problems. Physical examination revealed ulceration and swelling of all feet, mainly on the hindlimbs (Figure 1A). The lesions were 1 week old. The other vital signs, including appetite, heart rate, respiratory rate, mucous membrane color and hydration status, were within the normal ranges. Wound dressing accompanied with amoxicillin–clavulanic acid administration was performed, which partly improved the lesions. Subsequently, a punch biopsy was performed at the edge of the ulcerative wound 3 days after the first visit. Ulcerative, pyogranulomatous and eosinophilic pododermatitis with nematodes was diagnosed histopathologically. The cat was treated with wound dressing and additional treatment with emodepsine/praziquantel (Profender® spot-on, Bayer); the skin lesions recovered within 3 days post-anthelmintic treatment and completely recovered after 2 weeks (Figure 1B).

The skin for the biopsy was fixed with 10% buffered formalin and embedded in paraffin wax. The paraffin-embedded specimen was cut into 4-μm thick slices and stained with haematoxylin and eosin (HE) for histopathological examination.

**Figure 2:**
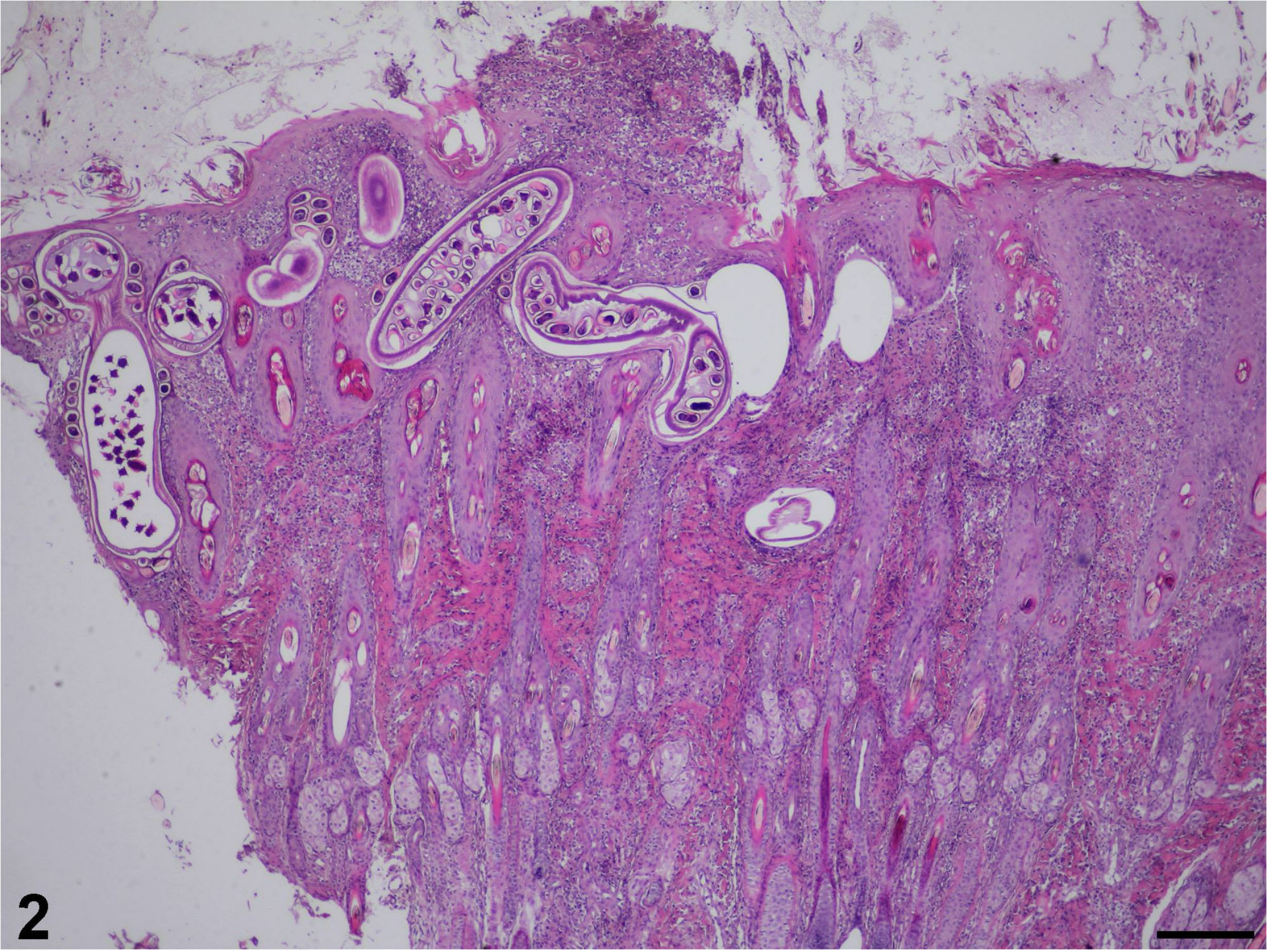
Histopathological section stained with haematoxylin and eosin revealing necrosis in the epidermis and nematode-like lesions in the dermis (200 μm scale bar).

**Figure 3:**
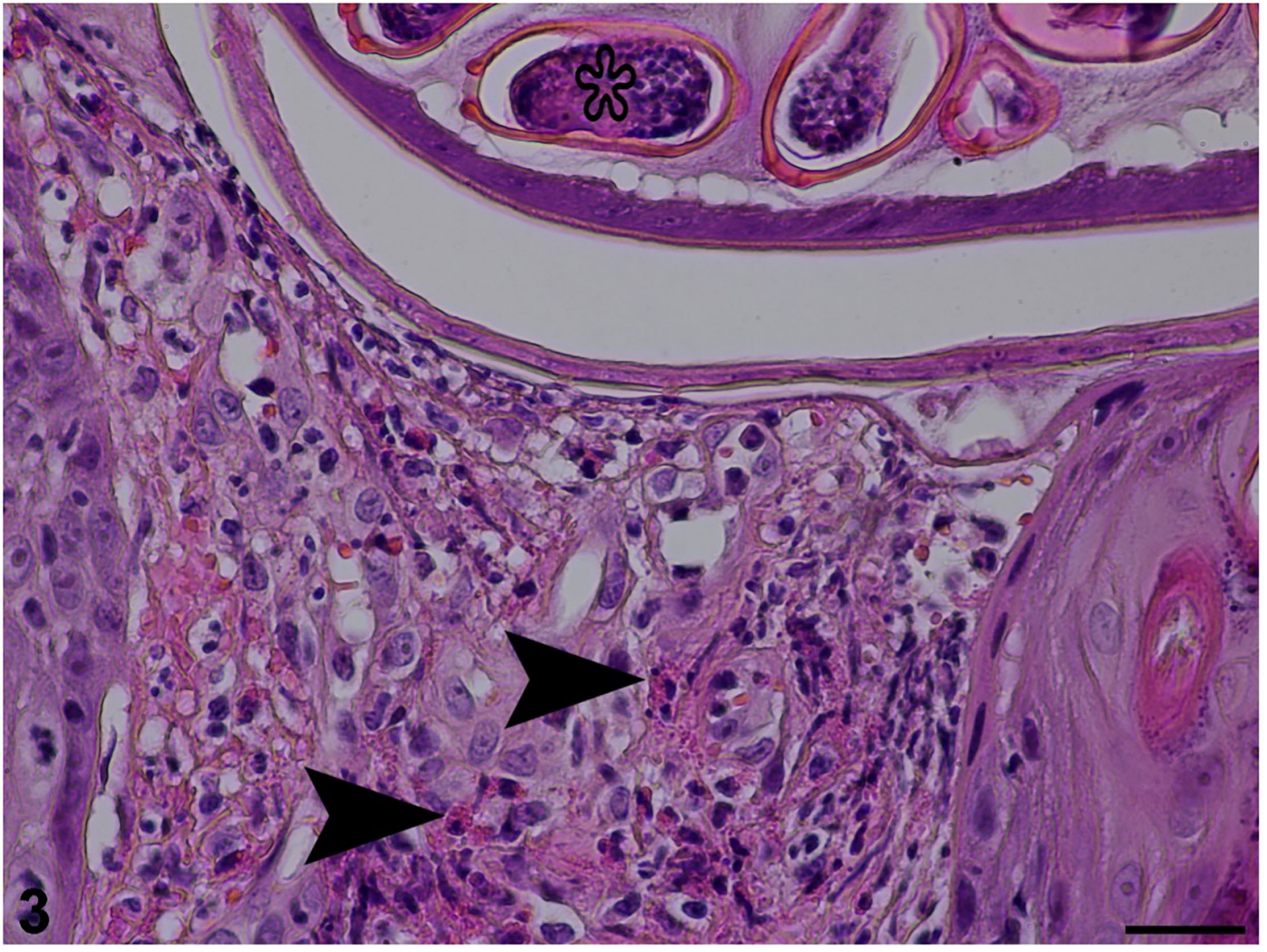
Histopathological section stained with haematoxylin and eosin revealing inflammatory cell infiltration (arrowheads) in dermis and egg (star) in the uterus of an adult female (25 μm scale bar).

Microscopically, the ulceration and some hyperplasia of the epidermis could be observed. The cross-section of the nematode was noticed in the epidermis and dermis (Figure 2). Figure 3 shows inflammatory cell infiltration. The nematode was 135–150 μm in diameter and characterized by a striated cuticle, hypodermal musculature, pseudocoelom, and a uterus containing eggs; eggs were also accumulated next to the adult. The egg was oval, 30 × 55 μm in size, with a bi-operculate thick wall (Figure 3). Infiltration of mixed inflammatory cells including neutrophils, macrophages and eosinophils was noted at the dermis, accompanied by necrosis (Figure 3).

**Figure 4:**
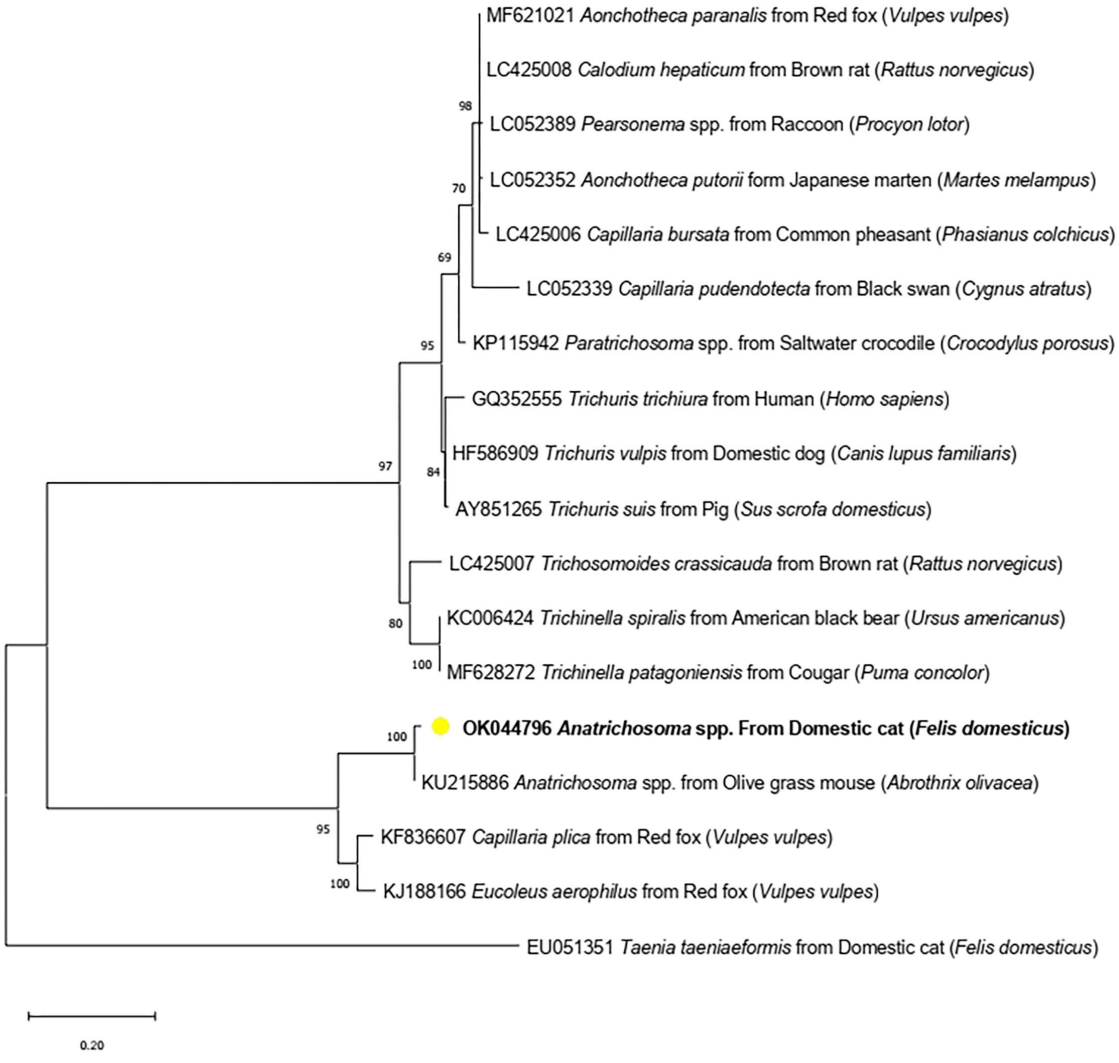
Maximum-likelihood phylogenetic tree showing the genetic relationship between *Anatrichosoma* spp. in cats (OK044796) (yellow dot) and other Trichinellida nematodes using *18S rRNA* gene.

Formalin-fixed paraffin-embedded samples were pooled and used for DNA extraction applying the QIAamp DNA FFPE Tissue Kit. The parasite species were identified *via* PCR targeting of the *18S rRNA* gene, using a customized primer, ANT-F (5′ATGGCCGTTCTTAGTTGGTG′3) and ANT-R (5′CGCTGA CGCTTTCAGTG TAG′3). Primers were designed based on the *Anatrichosoma* spp. *18S rRNA* sequence (KU215886) using Primer3 (version 0.4.0). The expected product was 213 base pairs. The PCR reaction mix contained 10 μl of 2 × ViRed Taq Master Mix (Vivantis, Malaysia), 0.5 μM of each primer and 4 μl of DNA. The conditions were initially 94°C for 5 min for pre-denaturation and followed by 35 cycles at 94°C for 30 s, 55°C for 30 s, 72°C for 30 s and final extension steps at 72°C for 10 min. The PCR products were run with gel electrophoresis and submitted to sanger sequencing. *Anatrichosoma* spp. DNA (positive control) was not used on PCR amplification while distilled water was used as the negative control. However, PCR was conducted twice separately and both expected products were submitted to sequencing. The results were identity. The sequence was aligned using BioEdit software (version 7.0.5.3) with several species of the order Trichinellida submitted in GenBank (https://www.ncbi.nlm.nih.gov/). A phylogenetic tree from the sequence of partial *18S rRNA* was constructed employing Maximum likelihood methods using the Kimura 2-parameter for the substitution model. The phylograms were statistically tested using a Bootstrap method with 1,000 replications. *Taenia taeniaeformis* (EU051351) was set as an out group. The phylogenetic tree was produced using MEGA X software and constructed using fragment *18S rRNA* sequences of several species of the order Trichinellida. The sequences were grouped into three clusters: *Trichuris*/*Capillaria* spp., *Trichinella* spp. and *Anatrichosoma* spp. (Figure 4).

The *Anatrichosoma* spp. obtained from this study (Accession number OK044796) has 99.00% identity with *Anatrichosoma* spp. from the olive glass mouse (*Abrothrix olivacea*) (KU215886). *Anatrichosoma* spp. found in this study (OK044796) was placed in the *Anatrichosoma* spp. cluster, which included *Anatrichosoma* spp. from the olive glass mouse *(Abrothrix olivacea)* (KU215886) and both *Capillaria plica* and *Eucoleus aerophilus* from the red fox *(Vulpes vulpes)*.

## Discussion

This study reports the first case of *Anatrichosoma* spp. infection in a cat in Thailand, confirmed by both pathological findings and molecular identification. Histological investigation showed numerous inflammatory cells infiltrated around the migratory tract, in accordance with previous reports, particularly adult and egg morphologies of the nematode (12). Nematode eggs were also found, with characteristics of *Capillaria*-like eggs operculated on both ends. *Anatrichosoma* spp. should be listed as one of the causative agents of pododermatitis in cats (12, 13). Pathological changes during infection included eosinophil infiltration, as previously described (12, 13). Adult *Anatrichosoma* spp. were typically found in the superficial layer of various mucosal epithelial tissues, including nasal, stomach, eye, skin and buccal cavities (5, 13, 16, 17). Female adults were commonly found in the superficial layer and males in the submucosal layer (13, 17). In this study, emodepsine/praziquantel (Profender® spot-on, Bayer) was used for the treatment of the feline anatrichosomiasis. In previous reports, feline anatrichosomiasis was treated with ivermectin at 0.3 mg/kg (12, 13).

Because the lifecycle of *Anatrichosoma* spp. is unclear, the transmission and reservoir host are poorly known. However, *Anatrichosoma* spp. commonly affects non-human primates (6, 7, 11, 18–22), suggesting that such primates are reservoir hosts for *Anatrichosoma* spp. Interestingly, this cat was raised in a suburban area and had no contact with any wild non-human primates. *Anatrichosoma* eggs were presented in ulcerative wounds. The eggs might contaminate the soil, and transmission *via* contact with contaminated soil should, therefore, be considered. Because *Trichuris trichiura* eggs have been reported in oral mucosal lesions in a child in Brazil (23), it has been suggested that *Anatrichosoma* spp. should be in the differential diagnosis list when *Capillaria*-like eggs were found in superficial cutaneous and subcutaneous sites from these animals (24). None of these capillarids can cause cutaneous lesions, whereas *Anatrichosoma* spp. can lead to cutaneous infections in pets and wild species (11–15).

This study revealed the sequence of an *18S rRNA* fragment of *Anatrichosoma* spp. in cats, suggesting that *Anatrichosoma* spp. in cats are genetically closely related to the nematodes of carnivores, *Capillaria plica* and *Eucoleus aerophilus*. Two related nematodes might share a genetic relationship within carnivore hosts. However, this cluster is separated from other *Trichuris* and *Capillaria* spp. of carnivores, such as *Trichuris vulpis*. Further molecular identification is, therefore, necessary. Unfortunately, the DNA sample from this study was obtained from formalin-fixed paraffin-embedded tissue, making it difficult to amplify a large fragment of targeted genes. Formalin lowers DNA yields because it can cause DNA–protein cross-links (25). Further studies are required to establish its prevalence in animals and humans.

## Conclusion

Anatrichosomiasis is a neglected parasitic disease caused by *Anatrichosoma* spp. Clinical presentation depends on the parasite species and host. In non-human primates, parasites were found in mucosal and submucosal tissues of the nasal cavity. In contrast to cats, parasites were found in the epidermis and dermis of carpal and tarsal pads, causing ulcerative pododermatitis. The life cycle of *Anatrichosoma* spp. is poorly understood, and the transmission route is still unclear. We expect this first report of feline anatrichosomiasis in Thailand by pathological findings and molecular confirmation to generate further studies in this interesting research field.

## Data Availability Statement

The original contributions presented in the study are included in the article/supplementary files, further inquiries can be directed to the corresponding author.

## Ethics Statement

Ethical review and approval was not required for the animal study because this is a clinical case. Written informed consent was obtained from the owners for the participation of their animals in this study.

## Author Contributions

WJ, SK, and PT conceptualized and reviewed the manuscript. WJ, SK, TS, and PT collected data. All authors contributed to the article and approved the submitted version.

## Funding

This work was supported by the Special Task Force for Activating Research, Chulalongkorn University (STF 6401531001-1).

## Conflict of Interest

The authors declare that the research was conducted in the absence of any commercial or financial relationships that could be construed as a potential conflict of interest.

## Publisher's Note

All claims expressed in this article are solely those of the authors and do not necessarily represent those of their affiliated organizations, or those of the publisher, the editors and the reviewers. Any product that may be evaluated in this article, or claim that may be made by its manufacturer, is not guaranteed or endorsed by the publisher.

## References

[1] Pence DB Little MD. Anatrichosoma buccalis sp. n (Nematoda: Trichosomoididae) from the buccal mucosa of the common opossum, Didelphis marsupialis L. J Parasitol. (1972) 58:767–73. 10.2307/3278311 5057228

[2] Kinsella JM Winegarner CE. A field study of Anatrichosoma infections in the opossum, *Didelphis virginiana*. J Parasitol. (1975) 61:779–81. 10.2307/3279491 1165567

[3] Conrad HD Wong MM. Studies of Anatrichosoma (Nematoda: Trichinellida) with descriptions of *Anatrichosoma rhina* sp. n and *Anatrichosoma nacepobi* sp n from the nasal mucosa of *Macaca mulatta*. J Helminthol. (1973) 47:289–302. 10.1017/S0022149X00026584 4201609

[4] Spratt DM. *Anatrichosoma haycocki* sp. n (Nematoda: Trichuridae) from the paracloacal glands of *Antechinus* spp. with notes on Skrjabinocapillaria skarbilovitsch. Ann Parasitol Hum Comp. (1982) 57:63–73. 10.1051/parasite/1982571063 7081890

[5] File SK. *Anatrichosoma ocularis* sp. n (Nematoda: Trichosomoididae) from the eye of the common tree shrew, *Tupaia glis*. J Parasitol. (1974) 60:985–8. 10.2307/3278532 4436773

[6] File S Kessler MJ. Parasites of free-ranging Cayo Santiago macaques after 46 years of isolation. Am J Primatol. (1989) 18:231–6. 10.1002/ajp.1350180306 31964032

[7] Long GG Lichtenfels JR Stookey JL. *Anatrichosoma cynamolgi* (Nematoda: Trichinellida) in rhesus monkeys, *Macaca mulatta*. J Parasitol. (1976) 62:111–5. 10.2307/3279053 815529

[8] Pampiglione S Orihel TC Gustinelli A Gatzemeier W Villani L. An unusual parasitological finding in a subcutaneous mammary nodule. Pathol Res Pract. (2005) 201:475–8. 10.1016/j.prp.2005.04.008 16136755

[9] Eberhard ML Hellstein JW Lanzel EA. Zoonotic anatrichosomiasis in a mother and daughter. J Clin Microbiol. (2014) 52:3127–9. 10.1128/JCM.01236-14 24899034 PMC4136162

[10] Takenaka T Ueki H Hashimoto Y Hashimoto K Matsumoto S. A survey of the prevalence of *Anatrichosoma* sp. in nasal cavities of cynomolgus monkeys. Jikken Dobutsu. (1989) 38:93–6. 10.1538/expanim1978.38.1_93 2714386

[11] Breznock AW Pulley LT. Anatrichosoma infection in two white-handed gibbons. J Am Vet Med Assoc. (1975) 167:631–3. 1176358

[12] Noden BH Du Plessis EC Morkel C Tubbesing U Soni M. *Anatrichosoma* sp. in the footpads of a cat: diagnosis and pathology of Namibian case. Vet Parasitol. (2013) 191:386–9. 10.1016/j.vetpar.2012.09.018 23062581

[13] Ramiro-Ibanez F Winston J O'Donnell E Mansell J. Ulcerative pododermatitis in a cat associated with *Anatrichosoma* sp. J Vet Diagn Invest. (2002) 14:80–3. 10.1177/104063870201400119 12680653

[14] Lange AL Verster A van Amstel SR. de la Rey R. *Anatrichosoma* sp infestation in the footpads of a cat. J S Afr Vet Assoc. (1980) 51:227. 7241491

[15] Hendrix CM Blagburn BL Boosinger TR Logan RT Lindsay DS. *Anatrichosoma* sp infection in a dog. J Am Vet Med Assoc. (1987) 191:984–5. 3679996

[16] Jackson RK Motzel SL Corrigan JE. Diagnostic exercise: cutaneous lesions and unilateral hind limb swelling in a rhesus monkey. Lab Anim Sci. (1996) 46:444–7. 8872999

[17] Little MD Orihel TC. The mating behavior of Anatrichosoma (Nematoda: Trichuroidea). J Parasitol. (1972) 58:1019–20. 10.2307/3286614 5078588

[18] Choong SS Mimi Armiladiana M Ruhil HH Peng TL. Prevalence of parasites in working pig-tailed Macaques (*Macaca nemestrina*) in Kelantan, Malaysia. J Med Primatol. (2019) 48:207–10. 10.1111/jmp.12416 31025372

[19] Klaus A Zimmermann E Roper KM Radespiel U Nathan S Goossens B. Co-infection patterns of intestinal parasites in arboreal primates (proboscis monkeys, *Nasalis larvatus*) in Borneo. Int J Parasitol Parasites Wildl. (2017) 6:320–9. 10.1016/j.ijppaw.2017.09.005 29988805 PMC6031963

[20] Kouassi RY McGraw SW Yao PK Abou-Bacar A Brunet J Pesson B. Diversity and prevalence of gastrointestinal parasites in seven non-human primates of the Tai National Park, Cote d'Ivoire. Parasite. (2015) 22:1. 10.1051/parasite/2015001 25619957 PMC4306024

[21] Petrzelkova KJ Hasegawa H Appleton CC Huffman MA Archer CE Moscovice LR. Gastrointestinal parasites of the chimpanzee population introduced onto Rubondo Island National Park, Tanzania. Am J Primatol. (2010) 72:307–16. 10.1002/ajp.20783 20014274

[22] Thilakarathne SS Rajakaruna RS Fernando DD Rajapakse R Perera PK. Gastro-intestinal parasites in two subspecies of toque macaque (*Macaca sinica*) in Sri Lanka and their zoonotic potential. Vet Parasitol Reg Stud Reports. (2021) 24:100558. 10.1016/j.vprsr.2021.100558 34024374

[23] Brustoloni YM Chang MR Lyrio de. Oliveira AL, Silva de Alexandre A. Trichuris trichiura eggs found in oral mucosal lesions in a child in Brazil. Parasitol Int. (2009) 58:98–100. 10.1016/j.parint.2008.09.002 18848902

[24] Núñez FA. Trichuris, Capillaria or Anitrichosoma? Parasitol Int. (2010) 59:303. 10.1016/j.parint.2010.02.008 20188210

[25] Jeon HK Kim KH Eom KS. Molecular identification of Taenia specimens after long-term preservation in formalin. Parasitol Int. (2011) 60:203–5. 10.1016/j.parint.2010.12.001 21163367

